# Less is more: selection from a small set of options improves BCI velocity control

**DOI:** 10.1088/1741-2552/adbcd9

**Published:** 2025-03-17

**Authors:** Pedro I Alcolea, Xuan Ma, Kevin Bodkin, Lee E Miller, Zachary C Danziger

**Affiliations:** 1 Department of Biomedical Engineering, Florida International University, Miami, FL 33199, United States of America; 2 Department of Neuroscience, Feinberg School of Medicine, Northwestern University, Chicago, IL 60611, United States of America; 3 Department of Physical Medicine and Rehabilitation, Feinberg School of Medicine, Northwestern University, Chicago, IL 60611, United States of America; 4 Department of Biomedical Engineering, McCormick School of Engineering, Northwestern University, Evanston, IL 60208, United States of America; 5 Shirley Ryan AbilityLab, Chicago, IL 60611, United States of America; 6 Department of Rehabilitation Medicine—Division of Physical Therapy, Emory University, Atlanta, GA 30322, United States of America; 7 W.H. Coulter Department of Biomedical Engineering, Emory University Atlanta, Atlanta, GA 30322, United States of America

**Keywords:** neural decoding, cursor control, center-out, iBCI model (jaBCI), discrete velocity control, monkey iBCI, motor cortex

## Abstract

**Objective.:**

Decoding algorithms used in invasive brain–computer interfaces (iBCIs) typically convert neural activity into continuously varying velocity commands. We hypothesized that putting constraints on which decoded velocity commands are permissible could improve user performance. To test this hypothesis, we designed the discrete direction selection (DDS) decoder, which uses neural activity to select among a small menu of preset cursor velocities.

**Approach.:**

We tested DDS in a closed-loop cursor control task against many common continuous velocity decoders in both a human-operated real-time iBCI simulator (the jaBCI) and in a monkey using an iBCI. In the jaBCI, we compared performance across four visits by each of 48 naïve, able-bodied human subjects using either DDS, direct regression with assist (an affine map from neural activity to cursor velocity, DR-A), ReFIT, or the velocity Kalman Filter (vKF). In a follow up study to verify the jaBCI results, we compared a monkey’s performance using an iBCI with either DDS or the Wiener filter decoder (a direct regression decoder that includes time history, WF).

**Main Result.:**

In the jaBCI, DDS substantially outperformed all other decoders with 93% mean targets hit per visit compared to DR-A, ReFIT, and vKF with 56%, 39%, and 26% mean targets hit, respectively. With the iBCI, the monkey achieved a 61% success rate with DDS and a 37% success rate with WF.

**Significance.:**

Discretizing the decoded velocity with DDS effectively traded high resolution velocity commands for less tortuous and lower noise trajectories, highlighting the potential benefits of discretization in simplifying online BCI control.

## 1. Introduction

An intracortical brain–computer interface (iBCI) uses spiking information from simultaneously recorded neurons to infer, or ‘decode’, a paralyzed user’s motor intent so it can be realized by an assistive device physically [1–4] or digitally [5–10]. Decoders are typically built by regressing imagined or observed action against the simultaneous activity of recorded neurons [11–13]. Virtually all cursor-control iBCIs use *continuous velocity* decoders, mapping the neural activity into any 2D velocity command vector. We hypothesized that constraining decoded velocity commands to a small set of selections would improve iBCI control because the simpler repertoire of resulting cursor kinematics would be easier to learn, the discrete nature of the commands would act as a de-noising step, and stopping would be made simpler by including a zero-velocity selection. To test this hypothesis, we created the discrete direction selection (DDS) decoder, which uses neural activity to select from a small menu of cursor velocity commands. Using the jaBCI (a neural firing rate synthesizer allowing noninvasive, real-time, human-in-the-loop use of iBCI decoders [14]), we compared closed-loop performance of human subjects using DDS to three commonly used continuous velocity decoders: the velocity Kalman filter (vKF [15]), ReFIT [16], and the so called ‘direct regression decoder with assist’ [17] (DR-A, an affine map from neural activity directly to continuous velocity commands). We also compared iBCI cursor control performance by a monkey using DDS and a Wiener filter (WF) (another direct regression-based decoder).

The vKF [5, 11, 15, 18] estimates intended velocity through a compromise between (a) what it has seen the cursor do in the past, given the current cursor state (state transition model), and (b) how similar the recorded neural activity is to what was expected given the predicted cursor state (measurement model). Both the state and measurement models are calibrated by regression on reference activity, and together they output a command from the continuous velocity plane. The ReFIT decoder [7, 16] uses the vKF architecture but extends the calibration procedure. The two most salient changes are that (a) the state transition model predicts that the cursor obeys physical laws of movement with damping, and (b) the measurement model is calibrated using the cursor states we assume the user intends rather than the actual executed states, which include targeting errors and jitter. These enhancements were made in part to overcome the difficulty vKF users had stopping the cursor. DR-A linearly maps neural activity directly into continuous velocity commands [17]. This circumvents the need for inverse solutions predicting neural activity from cursor state (that arguably introduce speed biases and errors when generating stop commands [17]), which are required by the measurement models of vKF and ReFIT. A further ‘assisted calibration’ feature was introduced that averages the preprogrammed ‘training’ cursor trajectory with the decoded trajectory to help regularize the calibration.

The DDS decoder maps neural activity into a selection from a small menu of possible cursor velocity commands. Using the same training data as do the continuous velocity decoders, DDS fits a multinomial model that maps the neural activity into the probability the user is choosing each of the possible selections. We implemented DDS with nine possible selections: up, down, left, right (each with a slow and fast speed), and stop. We also included a post-prediction step that blended selections together based on their estimated probabilities, creating a smooth transition between selections. The structure of DDS was designed, in part, to simplify what is in essence a motor learning problem faced by iBCI users: the mismatch between decoded and actual intentions makes using an iBCI more like learning a new skill than it is having one’s mind read [12, 19–24]. Discretizing the command space reduces high-frequency jitter in the cursor trajectory because only neural activity changes that cross the selection threshold affect cursor velocity, which may make learning iBCI control easier. Importantly, because DDS is discrete in velocity, as opposed to directly selecting target positions [25, 26], subjects can still navigate the entire workspace. The question we addressed in this study was whether trading fine-grained continuous control for the simpler and less variable trajectories DDS produces improves online performance.

The invasiveness of iBCIs limits subject availability, making decoder assessments with rigorous sample sizes extremely challenging. To circumvent this challenge we used the jaBCI model, developed and validated in prior work [14], to compare cursor control performance across decoders. The jaBCI produces synthetic neural firing rates from real-time human finger motion that any iBCI decoder can use (as a stand-in for recorded cortical neural firing rates) to produce commands for standard iBCI cursor control tasks. The jaBCI model thus allows us to test closed-loop cursor control with large numbers of human subjects using different decoders.

In this study, we used the jaBCI to compare center-out cursor control performance in 48 able-bodied, naïve human subjects, each of whom used either the vKF, ReFIT, DR-A, or DDS decoders across four visits. DDS users had the top performance by a substantial margin. Consistent with a report in two monkeys [16], we found that (1) ReFIT outperformed vKF subjects and (2) ReFIT could bring the cursor to a stop within the targets while vKF subjects could not. As a comparison to the jaBCI results, a monkey used an iBCI with both the DDS and WF (a common dynamic linear filter) [27–29] decoders. In those experiments, DDS also substantially outperformed the Weiner filter across multiple sessions. These results together indicate that DDS can improve online performance compared to several continuous velocity decoder designs commonly used for iBCI cursor control.

## 2. Methods

### 2.1. Human subjects

The Florida International University IRB approved the protocol for this research, approval identifier IRB-17-0205-CR06. The research was conducted in accordance with the principles embodied in the Declaration of Helsinki and in accordance with local statutory requirements. All participants gave their informed consent to participate. Inclusion criteria were neurologically intact, able-bodied people ages 18–65, without injury affecting manual dexterity, whose hand could fit in the CyberGlove III (CyberGlove systems). Subjects were compensated $10/visit. A total of 56 subjects with no knowledge of the experiment were recruited. Six subjects were excluded post-recruitment: one for failing to complete all visits, two for having fingers too short to reach the distal glove sensors, and three due to technical errors during data collection. The remaining 48 subjects (24 females, 24 males, aged 18–42) participated in four visits within a 14 d period. However, the 2nd visit of a subject was omitted as the system had them use vKF rather than DR-A. On the first visit, subjects completed a brief demographic survey and were pseudorandomly assigned by computer algorithm to one of the four decoder groups. At the start of each visit subjects sat approximately 70–90 cm in front of the computer monitor (34 cm × 60 cm) and an experimenter helped them don the CyberGlove.

### 2.2. The jaBCI model

**Figure 1.**
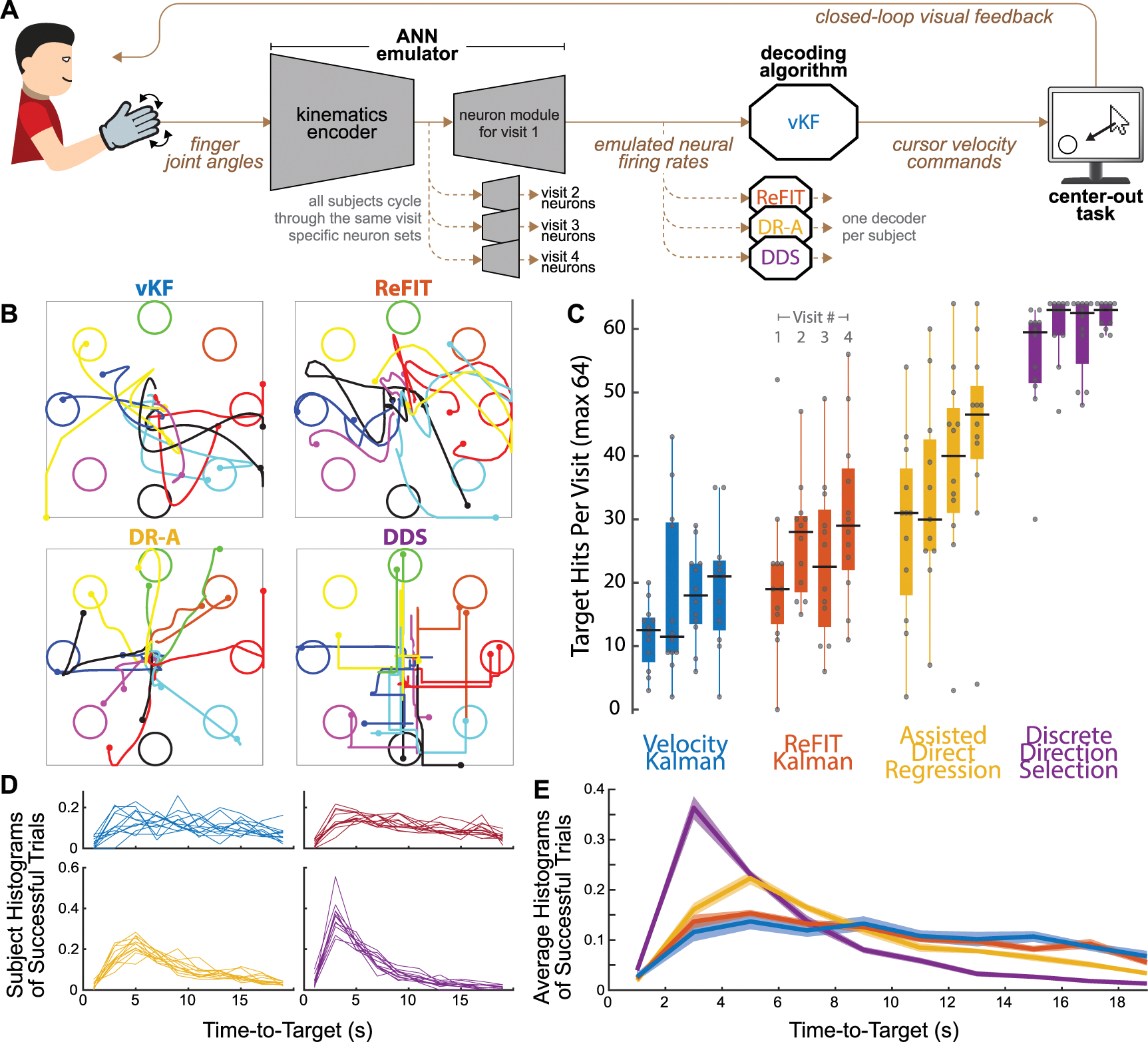
Cursor control performance of 48 naïve human subjects, each assigned one decoder for a series of four visits. (A) Schematic of the joint angle BCI (jaBCI) model of iBCI use. Human subjects’ finger joint angles were input to an artificial neural network (ANN) which output emulated neural activity. Emulated neurons were changed each time a subject returned for a new visit, and the decoders were recalibrated. Subjects performed a center-out target acquisition task in real time using decoding time bins of 50 ms. (B) Representative trajectories from successful trials in visit 4 from one subject in each group. Trajectories were truncated at 2.25, 2.25, 3.5, and 5.0 s after trial start from a visit with 17, 26, 42, and 59 total target hits for vKF, ReFIT, DR-A, and DDS decoder groups (i.e. typical success rates for each group). More time was included in DR-A and DDS to capture fuller trajectories because subjects moved more slowly overall (see also figure 2(B)). (C) Boxplots of number of targets hit in the center-out task out of 64 possible, outer grouping by decoder used (color) and inner grouping by visit number. Performance increased with repeated visits and also varied with the decoder. Dots are individual subjects, black bars are the mean, solid box is the interquartile range, and whiskers extend to 1.5 s.d. ± of the mean. (D) Trial time histograms for each subject (successful trials only, including the 0.5 s hold time, 2 s bins). Time distributions were consistent across subjects. Intra-group variability was larger in groups where fewer targets were hit, in part because fewer samples were available. (E) Across-subject averages (shaded patches are ± s.d.) of the histograms in D show that groups with better center-out cursor control performance (C) hit targets more quickly (more distribution mass at lower times). Additional statistical testing using a one-way ANOVA found a main effect (*p* < 0.001) and a post hoc multiple comparison test using Tukey HSD found that all groups had significantly different mean time-to-target (*p* < 0.05), with exception to ReFIT and vKF (*p* = 0.43).

The jaBCI model and its validation using the vKF decoder is described in detail in [14] and illustrated schematically in figure 1(A). Briefly, a subject wears a CyberGlove III that monitors the relative excursions of 19 different hand and finger joint angles. The same pre-trained artificial neural network used in the jaBCI validation study [14] computes a set of emulated neural firing rates based on the preceding 100 ms of joint angle input data. To align with contemporary firing rate integration bin widths used in iBCI studies, we set the emulator to emit neural firing rates every 50 ms. At each time bin the subject’s decoder uses the emulated neural activity to compute the velocity of the computer cursor and the graphical display is updated accordingly. The joint angle tracking, neural emulation, and decoding all run in real-time. To mimic the phenomenon of neuron turnover in iBCI studies [18, 30], we used four separate neural readout modules (one for each visit) that emulated 71, 45, 70, and 82 neurons, thus requiring subjects to recalibrate their decoders at the start of each visit. Since we had previously found the effect of neuron set on performance was negligible, we selected the sets used by the most subjects in the validation study [14]. The jaBCI is a iBCI model compatible with any firing rate decoder or cursor control task.

### 2.3. Protocol for cursor control in the jaBCI model

The jaBCI emulated a different set of neurons each visit (supplementary figure 1(A)—such that all subjects cycled through the same four sets on subsequent visits); accordingly, the subject’s decoder was calibrated at the start of each visit (supplementary figure 1(B)) using a four target calibration protocol similar to that used by several iBCI studies [5, 11, 15, 16]. To do this, subjects made a fist posture that was comfortable for them and were then shown a picture of a hand posture in the center of the workspace and a corresponding target in one of the four cardinal directions. This was designed to mimic iBCI experiments that also use four calibration targets. Subjects were instructed to initiate a transition from the fist to the displayed posture when the target changed color, following a computer cursor traveling via minimum jerk trajectory between the workspace center and the target (i.e. a ‘training cursor’) [18]. They were instructed to complete the transition in 1.2 s, just as the training cursor came to rest in the target. The four targets were presented in sequence and the sequence was repeated twice, consisting of one block, then the decoder was recalibrated using the data from the current and all prior blocks. Users performed 7–9 blocks. In blocks 1 and 2 only the training cursor was displayed, while in all subsequent blocks the decoded cursor was also displayed. Individual calibration trials were flagged as outliers automatically, using PCA through time to project each trial to a point in a 6-dimensional latent space. Each 6D projected point was labeled according to the calibration target displayed during the trial, and we determined each trial’s similarity to other trials to that target by computing its silhouette score [31]. Trials were flagged as an outlier (and removed from the decoder training data) if the silhouette score was less than zero, i.e. the given trial was more similar to trials from another calibration target than to its own. The silhouette score used in the outlier detection was based on the Euclidian distance between all pairs of points in the latent space of the given visit, clipped at the 90th percentile, which prevented extreme outliers from unduly influencing cluster centroids. If subjects did not have at least four non-outlier trials per target after 7 blocks, additional calibration blocks were added automatically one at a time until the four-trial condition was met (no more than two such blocks were ever added). If subjects began their hand posture transition prior to being cued, did not initiate the transition within 0.5 s of the cue, or completed the transition more than 0.25 s prior to the training cursor coming to rest in the target, a corresponding error message was displayed and that trial was repeated automatically (and data from the trial with the error was discarded).

After calibration, subjects freely explored an empty workspace with their cursor for 2 min before beginning a center-out target acquisition task (supplementary figure 1(C)). A target appeared at the workspace center that subjects were required to reach and stay inside continuously for a 0.5 s hold period; we define a target ‘hit’ as meeting this continuous reach and hold requirement, not merely the cursor reaching the target. The cursor was displayed as a dot approximately 25% of the area of the target, but only the cursor center was considered when determining target hits. Once the center target was hit, one of eight radially distributed peripheral targets was pseudorandomly displayed such that the order was not predictable and all targets appeared eight times (for a total of 8 targets × 8 occurrences = 64 targets). Subjects then had 20 s to hit the peripheral target. After the 20 s timeout period or target hit, the central target reappeared and subjects were again required to reach and hold there to trigger the next trial, for which there was no timeout duration. After 150 min the task ended, whether or not subjects attempted all 64 peripheral targets, which happened 12 times in the 191 subject visits. The monkey iBCI study (described in 2.7) used the same 4-target calibration and 8-target evaluation task, although with different numbers of trials and target size.

After the center-out task, subjects engaged in a typing task in which they were required to move and hold their cursor on keys displayed as a virtual keyboard to type target phrases (supplementary figure 1(D)). To register as a keypress, the cursor needed to stay continuously within the key for 1 s. A phrase would appear on the screen and subjects were instructed to type it as closely as possible within 2 min. The five phrases were (‘Hello World’, ‘Cyber Glove’, ‘Potato Chips’, ‘Neuroscience’, and ‘Game Over’). Scoring was calculated as the Levenshtein distance (LD), i.e. how many character deletions, insertions, or substitutions needed for the spelled phrase to match the given phrase of a trial. Values were then converted into a percent score, S=100·(LD–c)/c, where c is the total number of characters in the target phrase.

The workspace for the calibration, center-out, and keyboard tasks was a square with side length 2 a.u. (arbitrary units) centered at the origin, occupying 28.2 cm^2^ on the computer monitor. For the calibration (4 targets) and center-out task (8 targets), circular targets with a 0.15 a.u. radius were displayed at 0.85 a.u. from the the origin. The virtual keyboard had three rows of 10 square keys each, with a key side length of 0.198 a.u. that allowed a 0.01 a.u. gap between the edges of the keyboard and workspace boundary. The keys were laid out in a regular grid in standard QWERTY format, with the addition of ‘space’ on row two and two ‘delete’ keys and a ‘.’ key on row three. We enforced that the cursor always remained inside the workspace boundaries by clipping all x and y positions. (supplementary figure 1—includes a diagram of the experiment protocol and examples of all 3 tasks).

### 2.4. Decoders

The five decoders used in this study generated cursor velocity commands every 50 ms. We chose 50 ms because of its common use in prior studies [32–36]. There is evidence that KF and regression decoders perform well near this value [34, 35], and lower bin widths may violate spike count Gaussianity assumptions [25, 32–34, 36, 37] and propagate noise into the velocity predictions. The vKF, the recalibrated feedback intention-trained Kalman filter (ReFIT), and DR-A calibration decoders were used only in the jaBCI model. The WF decoder was used only in the monkey-controlled iBCI. The DDS decoder was used in both the jaBCI and monkey iBCI experiments, with the same available selections and mixing value (see 2.4.5).

#### 2.4.1. The vKF

The vKF decoder updated cursor velocity at every time bin, t, according to

(1) $$x_{t}=Ax_{t–1}+K\left(z_{t}–HAx_{t–1}\right)$$

where x is the cursor state vector containing the positions, velocities, and offset, ${\left[\begin{array}{lllll} p_{x} & p_{y} & v_{x} & v_{y} & 1 \end{array}\right]}^{T},\;z$ is the c-by-1 vector of neural firing rates (where C is the number of emulated neurons), A is the state transition model that predicts the next cursor state from the current state, H is the measurement model that generates the neural firing rates we expect given the state prediction, and K is the Kalman gain that weights the measurement estimates against the state estimates when creating the final update. The cursor position at t is set as the integrated velocity command produced by equation (1), not the filter-predicted cursor position.

Terms A,H,K, the state transition noise matrix, the measurement model noise matrix, and the estimate error covariance were computed as described in [15]. A and H were computed by regression on data from all non-outlier calibration trials, for A by relating the cursor state $x_{t–1}$ to $x_{t}$ and for H by relating $x_{t}$ to $z_{t}$. The transition and model noise matrices were estimated using A and H and the non-outlier calibration data. The Kalman gain and estimate error covariance matrix were updated adaptively by the Kalman filter equations and converged quickly during calibration. After the calibration task, these matrices were frozen, effectively creating a constant gain Kalman filter for the center-out task [15, 16].

#### 2.4.2. ReFIT Kalman filter

The ReFIT decoder, described in detail elsewhere [16], also applies cursor updates using equation (1); however, it computes A,H, and K differently. Briefly, the regressions for A and H use inferred rather than actual cursor state data, x. That is, cursor velocity is assumed to be pointing toward the target and have zero speed when in the target regardless of the actual decoded state, and the state transition model assumes integrated cursor velocity predicts its subsequent position. Second, when computing K from calibration data the uncertainty propagated through each iteration is updated to reflect the fact that cursor position is known with complete accuracy (it is shown to the subject). This is achieved by setting to zero the position-related elements in the error estimate matrix in the standard Kalman update equations.

#### 2.4.3. Assisted direct regression

The DR-A decoder updated cursor velocity at every time bin, t, according to

(2) $$x_{t}=Bz_{t}$$

where z is the (c+1)-by-1 vector of neural firing rates, B is the 2-by-(c+1) matrix of regression coefficients mapping neural activity into cursor velocity (the additional row is for x and y velocity offsets), and $x_{t}$ is the cursor state vector representing its velocity, ${\left[\begin{array}{ll} v_{x} & v_{y} \end{array}\right]}^{T}$. The cursor position at t is set as the integrated velocity command produced by equation (2). B is computed by regression relating $z_{t}$ to $x_{t}$ on data from all non-outlier calibration trials and using all emulated neurons with individual $x_{t}$ with velocity at or above 0.03 [17]. Additionally, during calibration the decoded cursor velocity was averaged with the training cursor velocity $x_{t}^{\text{train}}$ to produce the final decoded velocity $x_{t}$ at each time according to

(3) $$x_{t}=\alpha x_{t}^{train}+(1–\alpha )\;Bz_{t}$$

where (starting in block 3 when the decoded cursor first became visible alongside the training cursor) α decreased in each block from 0.8 by 0.2 to 0 [17]. In cases where additional calibration blocks were added, α remained at 0 and all center-out and virtual keyboard trials had α=0.

#### 2.4.4. The WF

The WF decoder [29] updated cursor velocity at every time bin, t, according to

(4) $$x_{t}=Bz_{\tau }$$

where $z_{\tau }={\left[\begin{array}{lllll} z_{t} & z_{t–1} & \ldots & z_{t–\tau } & 1 \end{array}\right]}^{T},\;z$ is a 1-by-c vector of neural firing rates, and the subscript denotes how many time bins in the past those firing rates occurred, with t being the current time. Thus, $z_{\tau }$ is a concatenation of the firing rates of the c neurons across τ instants into the past and an offset term. As with DR-A, $x_{t}$ is the cursor state vector representing its velocity, ${\left[\begin{array}{ll} v_{x} & v_{y} \end{array}\right]}^{T}$, which gets integrated to determine the cursor position. Here B is the is the 2-by-(cτ+1) matrix of coefficients mapping neural activity and its history into cursor velocity, which is computed by regression relating $z_{\tau }$ to $x_{t}$ on data from all calibration trials. In the monkey iBCI task we used τ=8, allowing WF to access firing rates up to 400 ms in the past. WF is like DR-A in that it is a single linear transformation mapping neural activity to cursor velocity with offsets to model baseline firing rates. It is dissimilar in that it includes 400 ms of firing rate history when decoding velocity; however, like all decoders used in the jaBCI and monkey iBCI, the decoder generated commands every 50 ms. Assisted calibration [17] was not used in the monkey iBCI experiment. We chose the WF because it is effective and actively used by the Northwestern team (e.g. [33, 37–39]), giving us a use-case comparative evaluation of DDS.

#### 2.4.5. The DDS decoder

The DDS decoder updated cursor velocity at every time bin, t, according to

(5) $$x_{t}=\xi \left(\frac{e^{Bz_{t}}}{\sum e^{Bz_{t}}}\right)$$

where $x_{t}$, the current cursor state vector representing its velocity, ${\left[\begin{array}{ll} v_{x} & v_{y} \end{array}\right]}^{T}$ and $z_{t}$ is the (c+1)-by-1 vector of neural firing rates (the additional row is for the offset term). B is the η-by-(c+1) matrix containing the coefficients mapping the current neural activity into the multinomial logistic regression model probability estimates for each of the η selections. Meaning, at each time bin the multinomial model produces a set of η probabilities, $p_{t}^{\left(i\right)}$, that represents its confidence that $z_{t}$ corresponds to each of the η selections (the sum in equation (5) runs over these η selections). Rather than choosing the velocity command with the multinomial model’s highest probability (winner take all), we performed a weighted average across the velocity commands of the most probable selections. The weighting was determined by a linear piecewise function of their probabilities, $\xi \;\left(p_{t}\right)$,

(6) $$w^{\left(i\right)}=max\left(0,\;min\left(1,\frac{p_{t}^{\left(i\right)}+\gamma –1}{2\gamma –1}\right)\right)$$

(7) $$\xi \left(p_{t}\right)=\sum_{i=1}^{n}v^{\left(i\right)}\cdot \frac{w^{\left(i\right)}}{\left\|w\right\|}$$

where $p_{t}={\left[\begin{array}{llll} p_{t}^{\left(1\right)} & p_{t}^{\left(2\right)} & \ldots & p_{t}^{\left(\eta \right)} \end{array}\right]}^{T}$ is the η-by-1 vector of multinomial model probabilities for each selection at time $t,\;v^{\left(i\right)}$ is the velocity command of selection i, and γ∈(0.51] is a scalar ‘selection mixing’ parameter. Equation (6) states that selections with probabilities below 1–γ do not contribute to the average, a selection with probability greater than γ completely determines the result and forces all other weights to zero, and all selections with probabilities in between are weighted linearly in proportion to the magnitude of their probability. Equation (7) states that the vector of selection weights, $w^{\left(i\right)}$, is renormalized to unity, then used in the weighted average of the velocity selections. γ=1 produces an average weighted by the direct multinomial model probabilities, and as γ approaches 0.5 then $\xi \left(p_{t}\right)$ approaches a winner take all weighting. This formulation preserves the DDS philosophy of selecting from a short list of possible velocity commands, while also providing users with some visual feedback about when $z_{t}$ nears a region in ‘neural activity space’ that is close to a boundary between velocity command selections. We used γ=0.85 and the η=9 velocity selections [0 1], [0, 0.5], [1, 0], [0.5, 0], [0, −1], [0, −0.5], [−1, 0], [−0.5, 0], and [0, 0], where each corresponds to $\left[\begin{array}{ll} v_{x}, & v_{y} \end{array}\right]$ in arbitrary workspace units per second.

DDS decoder calibration was done using the same task as all other decoders. To prepare the data for computing B, each trajectory from non-outlier trials was partitioned into fifths. Data from the beginning fifth of each trial was labeled as the ‘stop’ selection, [0, 0]. Data from the middle fifth of each trial was labeled as the ‘fast’ selection (1 unit s^−1^) in the direction of the target (e.g. [0, 1] for the North target, [1, 0] for the East target, etc). Data from the second, fourth, and last fifth of each trial was labeled as the ‘slow’ selection (0.5 units s^−1^) in the direction of the target. After labeling, the multinomial logistic regression model, B (not including $\xi \left(p_{t}\right)$), was fit using the scaled conjugate gradient descent algorithm. The available selections, γ value, and mixing equation were the same jaBCI and the monkey iBCI experiments.

### 2.5. Analysis of human subject jaBCI cursor control

We used a full factorial ANOVA *F*-test design to test the effects of visit number and decoder on task performance, as well as any interactions between them. Post hoc tests were done using the Tukey HSD method. To control for learning to estimate the effect of neuron set on enter-out performance we fit a line to the number of target hits across visits, separately for each decoder group. We then subtracted this trend off each visit’s average target hits to remove the effect. All statistical analyses used a critical *p*-value of 0.05.

We compared the number of cardinal and diagonal target hits for each decoder with paired *t*-tests, yielding four tests with 48 samples each (12 subjects × 4 visits/subject).

When analyzing the number of targets hit (figure 1(C)) we considered only peripheral targets, not the return to the center target, which did not have a timeout period.

Time-to-target histograms were binned into 2 s intervals and normalized to unit area (figure 1(D)). Only successful trials were considered, creating a maximum at the 20 s timeout period. Group averages (figure 1(E)) were computed by averaging individual subject histograms in figure 1(D). The statistical test described in figure 1(E) compared the four decoder groups with a one-way ANOVA and a post-hoc multiple comparison using Tukey HSD. Each decoder group had a different number of time-to-target data points corresponding to the number of successful trials in that group.

**Figure 2.**
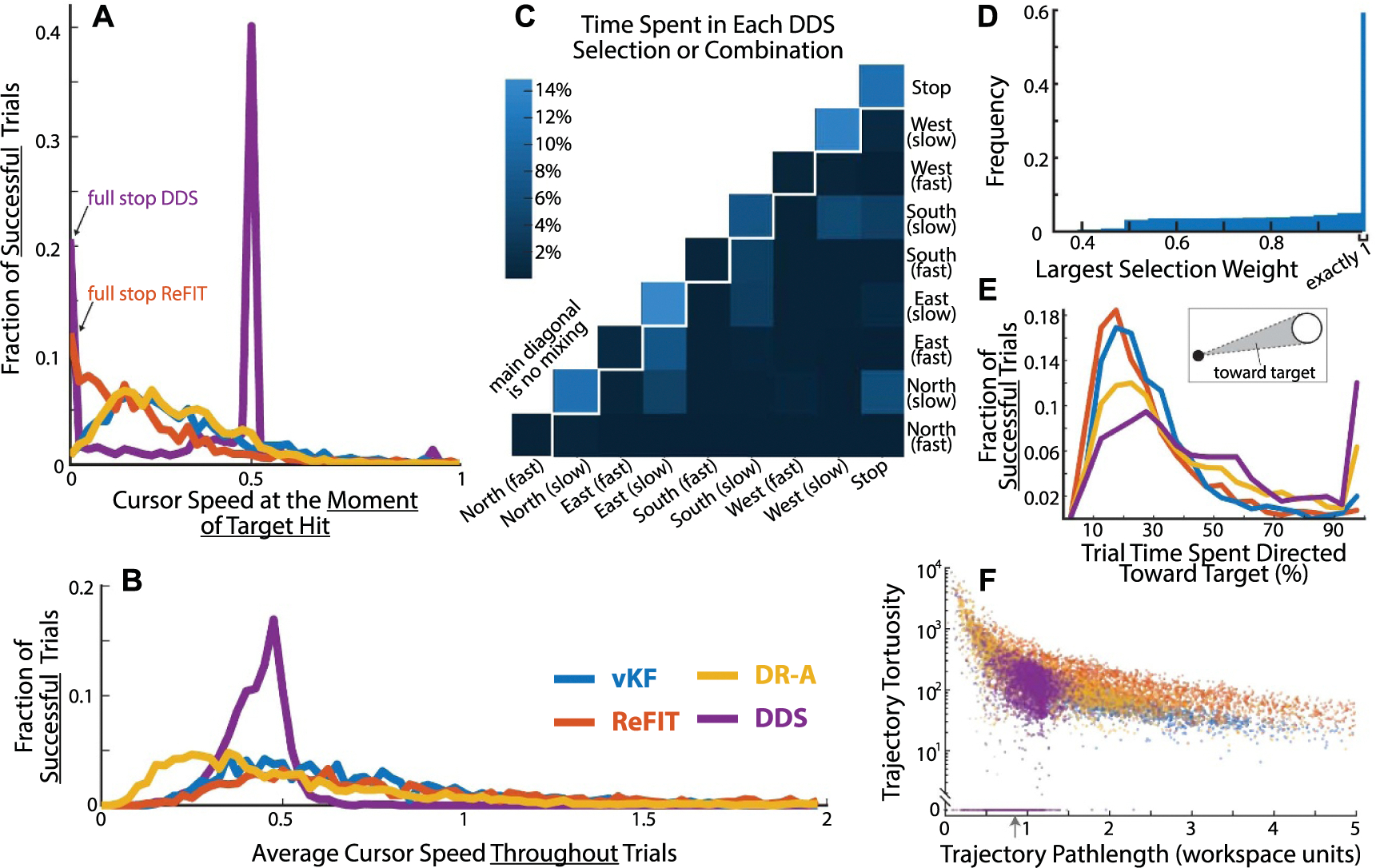
Synthesis of cursor control strategies across decoders. (A) The distribution of speeds at the moment of target hit. ReFIT and DDS users often brought the cursor to a full stop, while vKF and DR-A users did not. The DDS speed selections were 0.0, 0.5, and 1.0 units s^−1^, thus giving rise to the peaked distributions. (B) The distribution of average cursor speed throughout the entire trial shows DR-A and DDS groups have fewer high-speed trials. Histograms contain 804, 1208, 1710, and 2850 trials for vKF, ReFIT, DR-A, and DDS for panels A, B, and E. Speed was measured in workspace units per second, and the workspace was a square of side length two. (C) Time DDS subjects spent in each selection or combination. The four ‘slow’-only directions and ‘stop’ had the highest frequencies in the data. (D) The distribution of the largest selection weight shows subjects spent most of the time in a single selection (exactly 1). This panel shows the degree of mixing, whereas panel (C) shows whether any mixing occurred. (E) Histograms showing the percent of each trial the cursor spent heading toward the target (i.e. a velocity within the angle subtended by lines tangent to the target originating from the cursor, inset). (F) Trajectory pathlength versus trajectory tortuosity (integrated instantaneous curvature change normalized by pathlength) shows DDS subjects executed shorter and spatially simpler trajectories than other decoder groups. The occasional sharp turns in DDS trajectories generated tortuosity similar to the continuously smooth and simple arcs of vKF trajectories, although they were typically shorter. ReFIT trajectories contained the most erratic changes in direction; see also panel (E) and figure 1(B). The gray arrow denotes the straight-line distance from the workspace center to the center of the peripheral targets for reference. Datapoints are semi-transparent to facilitate visualization because many points overlap.

To analyze cursor stopping behavior we computed normalized cursor speed histograms using all individual trials as observations, not averages (figure 2). Figure 2(A) shows histograms of cursor speed at the exact moment that a target hit was registered, thereby capturing speed at a single 50 ms bin. There were no cursor speed requirements to register a target hit, only that the cursor center remain completely within the target for the full 500 ms hold time.

‘Dial in Time’, i.e. the time the cursor was within 2 radii of the target, was calculated for every trial and averaged for each subject visit. Subject-visit averages were compared using multiple independent *t*-tests, which compares all possible combinations between decoder groups.

The grid showing which DDS selections were mixed (figure 2(C)) is limited to showing only pairs of selections. Where more than two selections were mixed (≈7% of the time) the two selections with the largest weightings were counted. Data were pooled across all DDS subjects and visits (approximately 15 h of recordings).

We computed the time directed toward target measure (figure 2(E)) as the percentage of samples from a trial in which the direction of the decoded velocity was between the two rays emanating from the cursor position and contacting the target radius at the two opposing tangent points (see inset). All samples where the cursor was inside the target were counted as heading toward the target. All samples where the cursor was still in the home target from the previous trial were discarded, since often these involved heading corrections from the previous trial or reaction time. All successful trials were compiled into histograms of bin width 5% trial times and plotted separately for each decoder group.

Tortuosity, common in the vasculature literature [40, 41], was used as a measure of trajectory complexity and defined as the integrated instantaneous change in curvature, κ(t), normalized by total pathlength, L,

(8) $$T=\frac{1}{L}\int_{t_{1}}^{t_{2}}g\left(\frac{\;d\kappa (t)}{dt}\right)\;dt.$$

The curvature, κ(t), at each point is defined in the usual way as the reciprocal of the radius of the circle that best approximates the trajectory at each point. This measure is useful because it only penalizes direction changes if they happen with complex, continually changing arcs, meaning, there are ways to achieve low tortuosity besides straight-line trajectories. We take $g(x)\equiv \sqrt{\left|x\right|}$ to enforce positive values and maintain numerical stability when computing T for trajectories with sharp corners (a case not encountered in blood vessels). We take $t_{1}=0.25\;s$ and $t_{2}=3\;s$ into the trial to consider the initial part of the trajectory that consists approximately of the subject’s first attempt. The first 0.25 s often contained course corrections related to hitting the center home target at the end of the previous trial. We use at most 3 s of the trajectory to consider only the initial attempt at acquiring the target, and not the large corrections that would happen after the initial attempt failed (especially in the continuous velocity decoders), which would otherwise have inflated the tortuosity measures.

### 2.6. Correlations between calibration and cursor control outcomes in human subject jaBCI

We measured the difference between subjects’ decoded trajectories during calibration and the minimum jerk training trajectories shown during calibration to determine if it predicts closed-loop performance. Using each subject’s fully calibrated decoder from the center-out task, we decoded offline cursor kinematics from the emulated neural data they generated during the last 5 calibration blocks. Any automatically flagged calibration outliers were not used as part of the decoder calibration dataset, and were thus excluded here as well. We computed the discrete Fréchet distance between the decoded and training trajectories to produce a scalar dissimilarity measure. The discrete Fréchet distance is essentially the length of the longest line segment required to connect two points progressing along the paths through time. It is useful for trajectories because it does not harshly penalize similar shapes that are slightly out of phase. We then computed the correlation between the Fréchet distance for each subject-visit calibration and the number of targets they hit in the subsequent center-out task. Our inability to find a correlation between trajectory similarity and center-out performance was robust to many permutations of our analysis described above: (i) the number of calibration trials used (all trials versus only later trials), (ii) inclusion of outliers, (iii) use of pointwise root mean squared error as a trajectory similarity measure, (iv) correlating angular dispersion with center-out performance, (v) correlating the average silhouette score of how trajectories through time clustered in a 2D PCA projection, and others.

We also tested whether highly separable neural population dynamics predicts closed-loop performance. To measure separability, we projected the neural trajectories from all calibration blocks into the top three PCs defined by all non-outlier calibration trials [42]. We then computed the pairwise discrete Fréchet distances between all neural trajectories in the reduced space. The distance matrix was used to compute the silhouette score for each trajectory belonging to the cluster corresponding to the calibration target. The silhouette score is the normalized difference between the average distance of a trajectory to those in its nearest neighboring cluster and the average distance of that trajectory to all others in its own cluster. Thus, larger values indicate greater separation with (distance from) trajectories of neighboring clusters than its own cluster, making the data more separable. We then computed the correlation between the average silhouette score [31] for each subject-visit calibration and the number of targets they hit in the subsequent center-out task.

### 2.7. Protocol for monkey iBCI use

**Figure 3.**
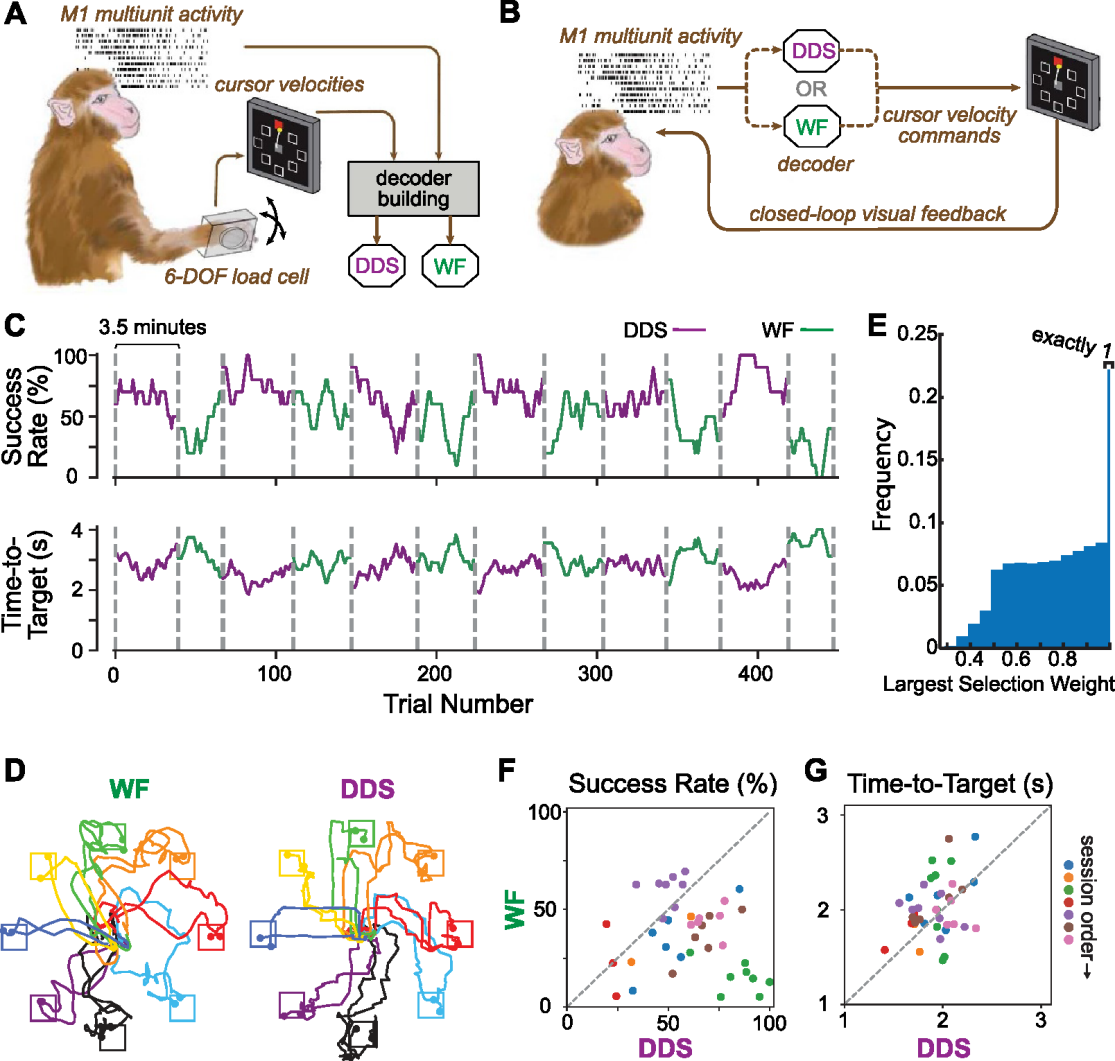
Cursor control performance of one monkey using two decoders. (A) Schematic of the decoder building process. A monkey was trained to move a cursor on a monitor by exerting 2D forces on a small box placed around its right hand. M1 multiunit activity and cursor velocities were synchronously recorded and used to build Wiener filter (WF)- and DDS-based decoders. (B) Schematic of the online closed-loop iBCI test. Once the decoders were built, the monkey was required to control the cursor on the monitor with M1 multiunit activity using either of the decoders. (C) Behavioral performance during a representative iBCI test session, as measured by success rate and time-to-target in causal 10-trial sliding windows. The two types of decoders were tested in interleaved 3.5 min blocks, as indicated by the vertical dashed lines. (D) Representative trajectories between the go cue and the delivery of reward from successful trials, two of which are plotted for each target. (E) Histogram of largest DDS selection weights over all sessions has a distribution qualitatively similar to that of the human jaBCI subjects (figure 2(D)), although spending somewhat less time in the purely unmixed selections. (F) and (G) Comparisons of success rate and acquisition time for the two decoders across 7 sessions (indicated by symbol color). Each dot represents the comparison between two adjacent blocks from a given session.

We used one 14 kg adult male rhesus monkey (Macaca mulatta) in this study, who had been previously trained, and implanted with a 128-channel ‘Utah’ electrode array (Blackrock Neurotech, Inc.) in the arm/hand representation of the left primary motor cortex (M1). The monkey performed an isometric wrist task requiring him to move a cursor from the center of the monitor to one of eight radial targets by exerting forces on a small box placed around his right hand (figure 3(A)). The box was padded to comfortably constrain the monkey’s hand and minimize its movement, and the forces were measured by a 6-DOF load cell (JR3 Inc, CA) aligned to the wrist joint. Flexion/extension force moved the cursor right and left respectively, while force along the radial/ulnar deviation axis moved it up and down. Each trial started with the appearance of a center target which the monkey was required to hold for a random time (0.2–0.5 s), after which one of the outer targets, selected in a block-randomized fashion appeared, accompanied by an auditory go cue. The monkey needed to move the cursor to the target within 2.0 s and hold the cursor within the target for 0.3 s to receive a liquid reward.

For each experimental session, M1 activity, and force were recorded using a Cerebus system (Blackrock Neurotech, Inc). The neural signals on each channel were digitalized, bandpass filtered (250–5000 Hz) and converted to spike times based on multi-unit threshold crossings (5.5 times the root-mean-square amplitude of the signal on each channel). We computed the spike counts in 50 ms, non-overlapping bins to obtain an estimate of firing rate as function of time for each channel.

All sessions used the four-target calibration and eight-target center-out tasks described in section 2.3. At the start of each session, we created a calibration dataset as the monkey performed the isometric wrist task in ‘hand-control’ mode (figure 3(A)) for 10 min. We used multiunit activity recorded from M1, along with cursor velocity to compute both WF and DDS decoders, using data from successful trials limited to the interval between the go cue and reward. To calibrate the DDS decoder, cursor velocities were divided into the same 9 selections as in the jaBCI study. After calibration, each session had multiple interleaved 3.5 min blocks in which either WF or DDS was used. We paired the success rate and acquisition time from temporally adjacent WF and DDS blocks and concatenated all pairs across the seven sessions to perform a two-sided paired *t*-test on 38 total sessions. Similarly, this was done within decoder groups as well, by comparing the success rate for cardinal and diagonal targets within WF and DDS.

All surgical and experimental procedures were approved by the Institutional Animal Care and Use Committee of Northwestern University under protocol #IS00000367 and are consistent with the Guide for the Care and Use of Laboratory Animals.

## 3. Results

### 3.1. Decoder comparison in center-out cursor control using the jaBCI model

The jaBCI uses a subject’s finger joint movements as input to an artificial neural network (previously trained on monkey M1 neural activity and human finger kinematics) to produce emulated neural activity in real-time (figure 1(A)). Any decoder can then translate that emulated activity into commands. Each subject here was assigned one decoder. To model the daily recalibration typically required in iBCIs due to changing neurons [5, 15, 43–45], at the start of each visit, subjects performed calibration posture transitions while a displayed ‘training’ cursor [18] followed a minimum jerk path to four cardinal direction targets. Subjects then performed an 8-target center-out task with small targets and a 0.5 s hold requirement designed to be relatively difficult to avoid performance ceiling effects (see discussion for comparisons to other iBCI studies). Representative cursor trajectories are shown from a subject in each decoder group on their last visit (figure 1(B)). DDS tended to generate more ‘staircase-like’ trajectories compared to the continuous velocity decoders.

The average number of targets hit differed between decoder groups, 16.7, 25.2, 36.1, and 59.4 for vKF, ReFIT, DR-A, and DDS (figure 1(C) and supplementary table 1 ANOVA *F*(3,175) = 131, *p* < 0.001, all post-hoc comparisons *p* < 0.002). There was no performance difference between the cardinal and diagonal targets, which is important for two reasons (supplementary figure 2, paired *t*-test pooled across visits and decoders *p* = 0.41, and when considering each decoder separately DR-A was the only decoder that reached significance, with a slight increase of 1.3 more diagonal targets hit per visit *p* = 0.009). First, the decoder training data were collected based on trajectories only to the cardinal targets, showing subjects could move to targets not explicitly in calibration. Second, the DDS decoder only had cardinal direction velocities available, implying that a limited set of selections does not prevent subjects from traversing the full workspace. A similar pattern of decoder performances held for a more challenging virtual keyboard typing task subjects attempted for the last 10 min of each visit (supplementary figure 3).

Subjects hit more targets on each subsequent visit despite the changing set of emulated neurons (slopes of linear fits across visits were 2.4-*p* = 0.08, 2.7-*p* = 0.06, 5.0-*p* = 0.12, 1.9-*p* = 0.11 hits per visit for vKF, ReFIT, DR-A, and DDS). The low learning rate for DDS was due to subjects reaching the performance ceiling. Although the emulated neurons changed each visit, subjects used the same four sets in the same order. This let us control for neural variability to compare directly between decoders in a way that an iBCI study cannot, as neuron selection is not under experimental control. After subtracting off the improvement due to learning across visits, there were no significant performance differences remaining between visits (supplementary table 2 ANOVA *F*(3,172) = 0.59, *p* = 0.70). This indicates that the set of neurons themselves did not have a strong influence on performance, agreeing with findings in our prior work [14].

Time-to-target was consistent among subjects within each decoder group (figure 1(D)). Although DDS had a maximum speed, unlike the continuous velocity decoders, DDS subjects reached targets faster (ANOVA *p* < 0.001). To measure stopping difficulty we used ‘dial-in time’, defined as the time the cursor spent within two target radii of the target center, excluding the 0.5 s hold. Dial-in times were 2.1 ± 0.6, 2.1 ± 0.5, 1.5 ± 0.4, and 1.1 ± 0.3 s for vKF, ReFIT, DR-A, and DDS (*μ* ± *σ*, all pairwise *t*-test comparisons were *p* < 0.001 except vKF-ReFIT with *p* = 0.57). Only subjects using the ReFIT and DDS decoders came to complete stops in the target (figure 2(A)), and vKF and DR-A subjects would slow down or circle within the target to satisfy the hold time requirement. DR-A subjects had the lowest average cursor speeds, and all the continuous velocity decoders had a long tail of trials with very high speeds where the cursor became difficult to control (figure 2(B)).

Figure 2(C) shows a histogram of the percent of time DDS subjects spent in each of the 9 velocity command selections. DDS mixes selections together when their estimated probabilities are close, which is displayed in the histogram as the intersection cell of each combination of selections, with the self-intersection along top diagonal representing the unmixed selections. DDS subjects spent approximately 60% of all trial time in unmixed selections. When selection mixing did occur, it was mainly between the ‘slow’ and ‘fast’ velocities in the same direction or between the ‘slow’ and zero-velocity selections (figure 2(C)), with only 16.7% of time spent in selections that were mixed between different directions. To produce the final velocity command DDS averaged all selections weighted by their estimated probabilities. To understand how strongly selections were mixed (not just if they were mixed at all, as in figure 2(C)), figure 2(D) shows the histogram of the largest selection weight at each moment. Because the weights across all selections sum to one, the greater the largest weight, the less mixing occurred. The far right of the histogram is when the largest weight was exactly one (i.e. fully unmixed selections, equivalent to summing the top diagonal cells in figure 2(C)), which was by far the most common. When mixing occurred, all combinations from equal mixing (largest weight 0.5) to near complete dominance by a single selection (largest weight 0.99) were equally likely. This is shown in figure 2(D) by the approximately uniform distribution from 0.5 (≈3.1%) to 0.99 (≈5.0%).

DDS Cursor velocities were oriented toward the target (within the angle formed by the pair of lines tangent to the two sides of the target) more often than other decoders (figure 2(E)) and DDS had substantially more trajectories that moved toward the target throughout the entire trial (sharp increase at the 100% bin). We evaluated cursor path simplicity using a tortuosity measure that integrates the instantaneous curvature derivative [40], such that simple constant curvature shapes (an arc of a circle or straight-line segment) have zero tortuosity. We found that ≈11% of all DDS trajectories were completely straight (figure 2(F), bottom points). The remaining trajectories had lower tortuosity and shorter pathlengths than ReFIT and DR-A. Trajectories from the vKF group had tortuosity similar to DDS (subjects tended to make smoothly varying constant curvature trajectories), but with longer pathlengths. Unlike the other decoder groups, DDS subjects rarely made very short trajectories that spiraled in on themselves (upper left) or simple but long trajectories (far right) caused by high velocities.

### 3.2. Decoder comparison in center-out cursor control by a monkey using an iBCI

To determine if the DDS performance translates from jaBCI to an iBCI cursor control, we compared the ability of a rhesus macaque to operate a brain-controlled computer cursor using a WF decoder and the DDS decoder. Following decoder calibration in each session, the monkey used the two decoders in interleaved 3.5 min blocks, with the session’s initial decoder determined randomly (figures 3(A) and (B)). Figure 3(C) shows the behavioral performance in a representative session. In most blocks, the monkey achieved a higher success rate and more rapid target acquisition with DDS than with WF. Notably, in the last four blocks, performance was maintained with DDS but dropped with WF. Figure 3(D) shows example cursor trajectories from successful trials. The ‘staircase’ structure in these trajectories was also present, but less exaggerated than that of the human jaBCI subjects (figure 1(B)), and correspondingly spent less time in unmixed selections (figure 3(E). Figures 3(F) and (G) compares the performance of the two decoders across all seven sessions. DDS had a higher success rate than WF in 31 of 38 pairs of temporally adjacent blocks and lower acquisition time in 26 pairs. The monkey achieved a higher overall success with DDS than WF (DDS: 61± 3% (mean ± s.e.), WF: 37± 3%, *p* < 0.001, two-tailed paired *t*-test). Time-to-target with DDS was slightly lower (DDS: 1.92 ± 0.03 s (mean ± s.e.), WF: 2.02 ± 0.05 s, *p* = 0.042, two-tailed paired *t*-test). Unlike in the jaBCI experiments, we found that the success rate for cardinal targets was slightly higher than that for diagonal targets for both DDS (cardinal: 63 ± 3% diagonal: 59 ± 4%, mean ± s.e., *p* = 0.02) and WF (cardinal: 40 ± 3%, diagonal: 34 ± 3%, *p* = 0.003) decoders. But the effect for both decoders was similar, suggesting that limiting selections to cardinal direction in DDS was not the primary driver of the performance increase on cardinal targets.

### 3.3. Offline analysis is not predictive of closed-loop performance in jaBCI

**Figure 4.**
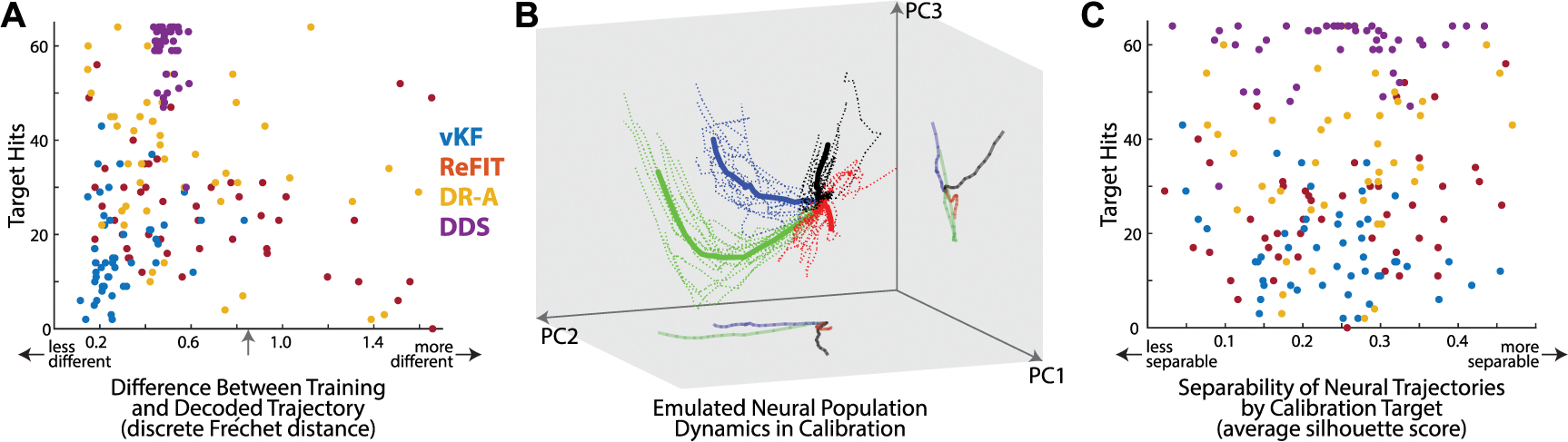
Accurate offline decoding in jaBCI was not predictive of closed-loop performance in the center-out task, nor was the low-dimensional separability of the emulated neural population dynamics. (A) There was no correlation between the difference of decoded and training trajectories (abscissa) and online control proficiency (ordinate). Trajectory similarity was measured with the discrete Fréchet distance, where zero indicates identical trajectories and grows with increasing dissimilarity. The gray arrow denotes the trajectory difference that would result if the decoded cursor had stayed at the origin throughout the trial for reference. (B) A typical example of emulated neural population dynamics during calibration from one subject visit. The low-dimensional space is defined by the top three PCs of all emulated neural activity during the calibration. Individual trials are dotted curves, colored by the calibration target for that trial, and solid traces are the spatial average over all trials to that target. ‘Neural trajectories’ typically formed distinct paths through PC space according to target and originate from the same location (waiting for the trial go cue). (C) We measured separability in terms of the silhouette score, which can range from −1 to 1, where larger values indicate that trajectories to a given target are increasingly similar to each other relative to trajectories to other targets. The separability of emulated neural population dynamics (average silhouette score for all trials in a subject visit, abscissa) was not correlated with online control proficiency (ordinate).

The jaBCI offline decoder accuracy was not predictive of closed-loop proficiency. Figure 4(A) shows there was no correlation between the average similarity of the calibration training cursor to the decoded trajectories and the subsequent number of hits in the center-out task (visits pooled across decoders *r* = −0.03, *p* = 0.72). This indicates that a decoder’s ability to reconstruct cursor kinematics of offline training data is not a key determinant of its success online.

The evolution of neural activity through neural state space during calibration trials was typically distinct for different targets (figure 4(B), projection onto the top three PCs capturing 97± 3% variance). However, the degree of separability was also not predictive of closed-loop proficiency. Figure 4(C) shows no correlation between the separability of projected neural population trajectories and the number of targets hit (visits pooled across decoders *r* = 0.08, *p* = 0.26). The lack of a relationship between neural trajectory separability and performance is surprising from a purely machine learning perspective, where accurate classification depends on being able to partition data in the feature space [46], as well as from a neuroscience perspective where some hypothesize that separability of low-dimensional neural population trajectories fosters more robust computation [43, 47, 48–50].

## 4. Discussion

The DDS decoder maps neural activity into a selection from a short list of velocity commands, and outperformed traditional continuous velocity decoders in the human-in-the-loop jaBCI model (N=48) and in iBCI control by a monkey. The result opens the door to a new type of decoder that exploits the accuracy of discrete selection [25] without sacrificing the flexibility of full workspace accessibility. The work also highlights the utility of the jaBCI, which can generate predictions about decoder performance that are statistically robust across subjects to screen ideas for follow-up in the more invasive and resource-intensive human or monkey cursor control iBCI studies.

Simultaneously decoding continuous cursor direction and speed is challenging. The stochasticity of neural activity leads to noise in decoded cursor velocity (and thus position), which degrades the ability to navigate to and hold the cursor still inside a target. Information about intended speed may also not be well represented in the cortical populations typically used in iBCI [44], making it harder still to decode intended stopping. ReFIT addressed this by setting velocity to zero (regardless of decoded velocity) whenever the cursor is inside a target during calibration [16], which allowed jaBCI subjects to stop consistently inside targets (figure 2(A)). The speed dampening KF decoder (not tested here) dynamically scales down the speed prediction in the KF state transition model when changing directions [44]. Some argue that affine transforms directly from neural activity to velocity commands, like DR-A and WF, improve cursor speed estimates over KF implementations that estimate neural activity from cursor states [17]. However, DR-A subjects did not stop the cursor (figure 2(A)), but DR-A did prevent many trials with high, and perhaps unmanageable, average cursor speeds (figure 2(B)).

Another approach has been to use two parallel decoders, one responsible for continuous velocity control and the other for a ‘neural click’ command. Decoding a discrete state propagates far less of the neural variability into cursor state noise because the binary decision will not often flip in response to small changes in the neural input. Clicks have been implemented with Bayes [8], LDA [45], or hidden Markov models [51] or by blending a zero-velocity command with the continuous velocity command in proportion to an LDA classifier’s confidence in the click state [52]. The continuous velocity decoder can even be abandoned altogether for accurate and efficient target selection from preset grids [25] or letters [26]; however, this approach forecloses the possibility of continuous control.

The premise behind DDS is to use the noise-rejection advantage of discrete classification to directly select cursor velocity while retaining the ability to traverse the entire workspace, since position is unconstrained. Unlike methods that use smoothing to denoise trajectories (e.g. long bin widths or temporal averages [53, 54]), DDS does not introduce lag because the velocity commands are issued every time bin. The noise rejection comes at the expense of coarsely decoded velocity (e.g. staircase-like trajectories, figures 1(B) and 3(D)), but this trade-off appears to benefit performance (figures 1(C) and 3(F)) and create simpler trajectories (figure 2(F)). The coarse-grained commands consequently led to below average offline accuracy (figure 4(A)) because discrete velocities could not reconstruct the continuously varying calibration trajectories (consistent with the observation that offline accuracy is not predictive of closed-loop iBCI performance [13, 54–60]). The extra time incurred when taking a staircase rather than straight trajectory to the target is small, so accepting coarse decoding for noise rejection appears to be a good trade-off in tasks where trial times longer than a second are permissible. For example, in our cursor task a staircase is 176 ms longer than a straight path to the target (assuming the slow velocity selection), <2% of the average trial time. In fact, subjects tended to spend more time in single, unmixed selections as their performance improved across visits (54%, 57%, 63%, and 67%), meaning they did not appear to avoid the staircase trajectories.

The selection mixing in DDS softens the boundaries between discrete velocity commands, which makes those regions more similar to continuous velocity control and thus permits more cursor noise. The amount of mixing can be tuned from winner-take-all, γ=1/2, to an average weighted exactly by the decoded probability estimates, γ=1. We used γ=0.85, but future studies could optimize γ to improve performance. Anecdotally, in internal testing, we found that γ=1/2 was difficult to control, perhaps because without any mixing there were no visual cues for when the decoder was about to switch selections, whereas with mixing there is a short and smooth transition between velocity commands as the neural activity approaches the classifier’s selection boundary. Optimizing other DDS parameters, such as the number of selections, the speeds of each selection, access to past neural activity, the classification model, or even the calibration protocol (e.g. adding separate calibration trials for different cursor speed or heading selections), may improve performance.

The reason for the counterintuitive performance gain that came from coarse-graining velocities is unclear. Speculatively, it may be that DDS effectively ‘teaches’ subjects to execute commands with coarse precision (e.g. more like ‘go left’ than ‘speed × cm s^−1^ at heading y degrees’). This may improve closed-loop control because decoding a selection from a small set of options can be done more accurately than can high-precision velocity commands, thereby eliminating the need to correct persistent errors in heading generated in continuous velocity decoding. In essence, DDS does not attempt to decode commands at higher precision than the neural activity supports. Future work could investigate how the supported precision (i.e. number of available selections) depends on channel noise or number of recorded neurons.

Parameter optimization may lead to changes in behavior as well. We found that the ‘slow’ speeds were greatly preferred by both human and monkey subjects. It could be that this was a strategy to improve cursor control (figure 2(B)), but it could also be that it was a consequence of assigning 3/5 of the calibration data to the ‘slow’ selection during model training (however, the ‘stop’ selection was assigned only 1/5 of the calibration data and was still frequently visited). Our current study cannot distinguish between these explanations.

A limitation of this study, and all iBCI studies, is that direct comparison of our results to other work is challenging because of the wide array of tasks, decoders, decoder parameterizations, calibration techniques, and levels of subject training used in the literature. We selected a group of commonly cited decoders and a common task to facilitate these comparisons; however, important questions remain that our study cannot address, such as whether shorter 15–20 ms bin widths [11, 61] rather than 50 ms [32–36] would shrink the performance gap with DDS, whether DDS would outperform modern deep learning decoders [34, 62], or how decoder performance depends on target size and position.

We corroborated two findings shown in iBCI studies: (1) ReFIT’s ability to improve stopping [16, 63] and (2) ReFIT’s better performance than vKF [16]. To our knowledge there have been no direct iBCI comparisons between DR-A (or DR-like decoders) and ReFIT, so DR-A’s better performance in the jaBCI is a prediction rather than a validation. We also found that DDS outperformed DR-A in the jaBCI and a similar direct-regression decoder (WF) in a monkey iBCI. This is strong evidence that cursor control decoders designed, tested, and found to perform well with the jaBCI will have correspondingly strong performance in iBCIs. jaBCI subjects using continuous velocity decoders hit fewer targets than what is often reported for iBCIs, in part because we used smaller targets (1.7% of workspace area rather than 9.3% [5], 5.7% [16], or 3.2% [18]) that were farther from the center (35% of the total workspace distance to the nearest target edge rather than 16% [5], 26% [16], and 32% [18]). In an iBCI task of comparable difficulty (1.2% area and 37% distance) [6], better performance was reported after 1000 d, as opposed to four in this study. In prior jaBCI validations we matched the iBCI task parameters precisely, and observed equivalent performance in jaBCI [14]. The jaBCI-emulated neural activity also matched firing rate statistics and low-dimensional population behavior of monkey M1 activity during reaching [14]. Here we opted for a more difficult task to prevent subjects from reaching a performance ceiling, which would compress differences between decoders, although DDS subjects appeared to reach this performance ceiling anyway (figure 1(C)).

These results demonstrate that an iBCI decoder using coarse-grained velocity commands can outperform continuous velocity decoders. Giving up the precision control of continuous decoding and accepting worse offline performance to achieve simpler low noise cursor trajectories appears to be a beneficial trade for online control. This reinforces the importance of closed-loop subject-decoder interactions in iBCI design and control.

## 5. Conclusion

We show, perhaps counterintuitively, that reducing precision in iBCI velocity decoding substantially improves cursor control performance. We speculate DDS achieves this improvement by leveraging the noise-rejection advantage of discrete classification, yet, unlike traditional discrete decoders, DDS can be used as flexibly as continuous velocity decoders because it retains the ability to reach anywhere on in workspace. We encourage other groups investigating BCI cursor control to test if DDS or its variants improve control in other contexts. In the future we plan to characterize the sensitivity of cursor control performance to each of the DDS parameters, to optimize them to further improve performance and to see how DDS compares to more modern decoders such as Unscented Kalman Filter [64] and neural networks [34, 62].

## Supplementary Material

Supplemental Figures and Tables

## Data Availability

The data that support the findings of this study are openly available at the following URL/DOI: https://doi.org/10.5061/dryad.wh70rxwx6.
