# Supplementary material for: Less is more: selection from a small set of options improves BCI velocity control
Source: J Neural Eng. Author manuscript; Available in PMC 2025 May 5. (PMC12051477; doi:10.1088/1741-2552/adbcd9)
Supplement: Supplemental Figures and Tables [file NIHMS2073720-supplement-Supplemental_Figures_and_Tables.pdf]

Supplemental Figures

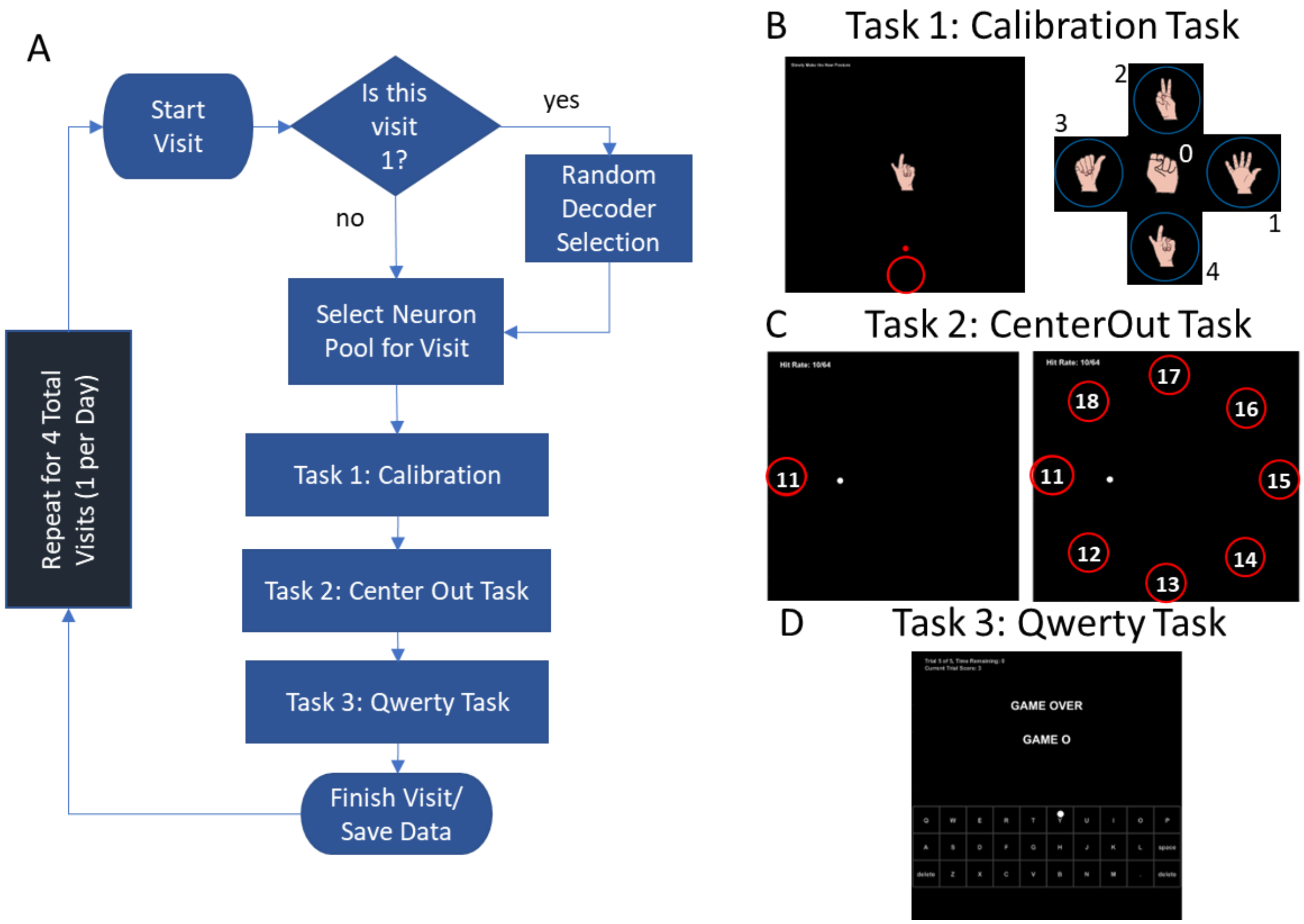

Supplemental Figure 1. Experimental protocol. A) Describes the protocol for a subject's 4 visits. In visit 1 subjects will be randomly placed into a decoder group, will be selected into the visit 1 emulated neuron set, and complete 3 tasks. In the following visits (held on different days) subjects will be placed into different emulated neuron sets and complete the same 3 tasks. B) Depiction of the calibration task, showing the four displayed postures and their corresponding target direction. C) Depicts the Center-Out task, by showing how a singular trail would look like if a subject got the left target in trial 11 (left-B), and showing all target locations that users will have to target throughout the 64 trials (right-B). Each target will be appearing 8 times throughout the task. D) Depiction of Qwerty Task, which involves subjects spelling out 5 words or phrases in 2 minutes or less each.

A)

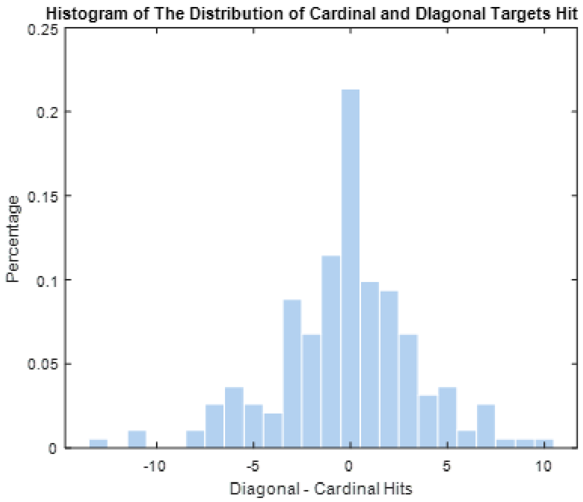

B)

Paired Samples Test

		Paired Differences							Significance	
					95% Confidence Interval of the Difference					
		Mean	Std. Deviation	Std. Error Mean	Lower	Upper	t	df	One-Sided p	Two-Sided p
Pair 1	Diagonal - Cardinal	-.21466	3.61590	.26164	-.73075	.30143	-.820	190	.206	.413

Supplemental Figure 2. Cardinal vs. diagonal target hit analysis. A) This histogram shows the distribution of the difference between diagonal targets and cardinal targets hit in the CenterOut task for all subject-visits. B) Analysis of histogram and additional statistical analysis with a two-sided p score of 0.413 showed that subjects had no preference in hitting cardinal over diagonal targets across all subject visits. This supports the use of only 4 directions during calibration.

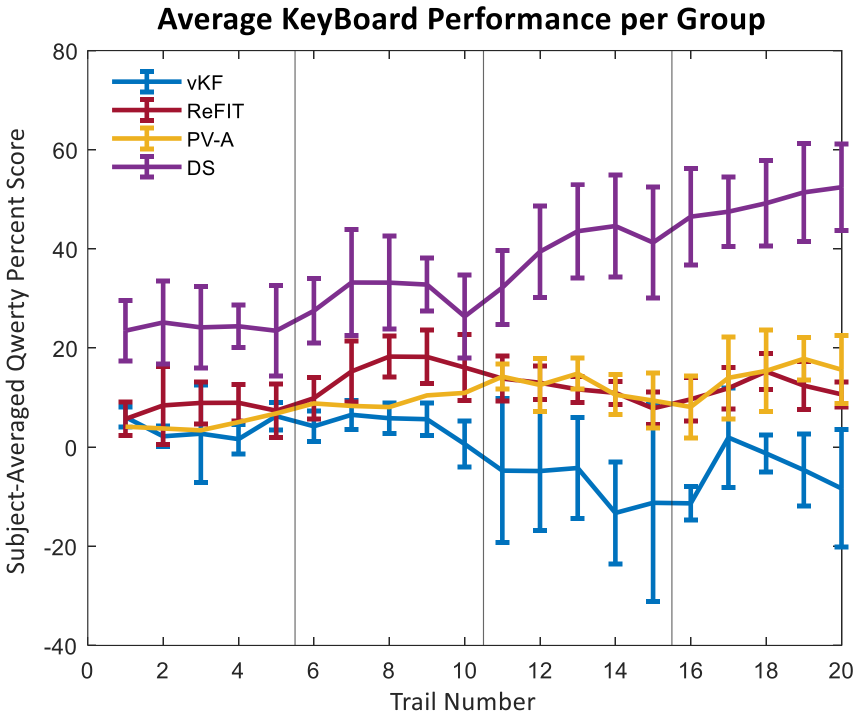

Supplemental Figure 3. Performance on the virtual QWERTY keyboard task. Each line is the mean percent score (y-axis) of every trail. Vertical lines denote transitions between visits. Error bars in each line represent the standard error of all subjects in a group. This task was considerably more difficult than the center-out task, with only DDS subjects able to meaningfully type (note scores of near zero for ReFIT and DR-A, and a negative score for vKF). Negative scores occur when the subject types more incorrect keys than correct ones, meaning vKF users would have performed had they not typed at all.

a. R Squared = .704 (Adjusted R Squared = .678)

Source	Type III Sum of Squares	df	Mean Square	F	Sig.
Corrected Model	52081.770[a]	15	3472.118	27.705	<.001
Intercept	225220.493	1	225220.493	1797.104	<.001
Groups	49119.354	3	16373.118	130.646	<.001
Visits	2429.418	3	809.806	6.462	<.001
Groups * Visits	521.774	9	57.975	.463	.898
Error	21931.727	175	125.324		
Total	299389.000	191			
Corrected Total	74013.497	190			

Supplemental Table 1. Statistical analysis of between group effects based on the results of the CenterOut task using the Full Factorial Anova F Test. Shows the effect of visits and the interaction between days and decoders. Results suggest that decoders and visits have a significant effect with a p < 0.001, while exhibiting no significant interaction. Post hoc analysis with Tukey HSD further revealed that all decoders are significantly different from one another and that only visits 1 and 4 are significantly different.

a. R Squared = .702 (Adjusted R Squared = .676)

Source	Type III Sum of Squares	df	Mean Square	F	Sig.
Corrected Model	51694.780[a]	15	3446.319	27.070	<.001
Intercept	166831.281	1	166831.281	1310.417	<.001
Groups1	51258.688	3	17086.229	134.208	<.001
Neurons	240.150	3	80.050	.629	.597
Groups1 * Neurons	185.046	9	20.561	.161	.997
Error	21897.591	172	127.312		
Total	240973.287	188			
Corrected Total	73592.371	187			

Supplemental Table 2. Statistical analysis of between group effects based on the results of the CenterOut task using the Full Factorial Anova F Test shows the effects of neuron set and the interaction between neuron set and decoder. The reason the tested effects change is due to the fact learning has been removed to focus on the effect of the different neurons used in each visit. Analysis revealed that there was no effect of neuron set on subject performance and that even when learning is removed the effect of decoders remained significant. Important value to consider is the neuron set p of 0.722, which is greater than 0.05 suggesting no significant difference of performance based on the neuron set.

ANOVA

	Sum of Squares	df	Mean Square	F	Sig.
Between Groups	51377.316	3	17125.772	140.546	<.001
Within Groups	22908.125	188	121.852		
Total	74285.441	191			

Supplemental Table 3. The table shows a One-Way Anova F-Test analysis testing the effect of decoder groups after removing all learning gained from subjects throughout the visits. This analysis helps show how the sum of squares between and within groups differ. Analysis shows that despite the large variance of subjects within groups, the difference between groups was larger.
